# Stromal Vascular Fraction Across Different Clinical Indications: A Pro-Regenerative *Fil Rouge*

**DOI:** 10.3390/jcm15082937

**Published:** 2026-04-13

**Authors:** Giuseppe Perale, Daniel Schmauss

**Affiliations:** 1 Faculty of Biomedical Sciences, University of Southern Switzerland, USI, Via G. Buffi 13, 6900 Lugano, Switzerland; 2 Ludwig Boltzmann Institute for Experimental and Clinical Traumatology, Donaueschingenstrasse 13, 1200 Vienna, Austria; 3 Department of Plastic, Reconstructive and Aesthetic Surgery, Ospedale Regionale di Lugano, Ente Ospedaliero Cantonale (EOC), 6900 Lugano, Switzerland

**Keywords:** stromal vascular fraction, adipose-derived stem cells, regenerative medicine

## Abstract

Stromal vascular fraction (SVF) has emerged as a versatile autologous therapeutic strategy across multiple regenerative medicine applications. Derived from adipose tissue, SVF exerts its effects primarily through paracrine, immunomodulatory, pro-angiogenic, and anti-fibrotic mechanisms rather than direct cell differentiation. Potential regenerative outcomes have been reported in bone, cartilage, scar modulation, and neural repair, highlighting a shared pro-regenerative cascade centered on early inflammation control and vascular support. Indeed, increasing evidence suggests that synergy with biomaterials and point-of-care one-step approaches further enhances SVF efficacy. Regarding the real clinical potential, however, transability is still limited due to heterogeneity in isolation methods, lack of standardization, and insufficient large-scale randomized controlled trials.

## 1. State of the Art

Stromal vascular fraction (SVF), the heterogeneous cell population obtained from adipose tissue comprising adipose-derived stem cells, endothelial progenitor cells, pericytes, macrophages, and other supporting cells, has emerged as a promising and versatile therapeutic tool [1,2,3]. Across diverse clinical indications—ranging from bone defects to cartilage damage, osteoarthritis (OA), avoidance of scar formation in plastic surgery, and spinal cord injury (SCI)—the properties of SVF consistently converge thanks to the combination of their secretome and the synergistic effect of the different cellular populations [4]:

1. Anti-inflammatory and immunomodulatory actions: SVF cells release anti-inflammatory cytokines (e.g., IL-10, TGF-β) that reduce pro-inflammatory mediators (e.g., IL-1β, TNF-α, MMPs) [5].
2. Angiogenesis: SVF contains endothelial progenitor cells and pericytes and secretes growth factors such as VEGF, which collectively support the formation of new blood vessels and improve vascularization in grafted or damaged tissue [2,6].
3. Anti-scar and anti-fibrotic effects: As SVF accelerates transition from inflammation to remodeling, this influences collagen fiber organization and reduces scar thickness, scar irregularity, pigmentation, vascularity, and itching [7].

As one of the most common forms of regenerative medicine, historically, bone regeneration was one of the earliest translational successes of SVF use in humans, especially in oral/maxillofacial applications (e.g., maxillary sinus floor elevation, alveolar ridge augmentation) and in translational studies on preclinical models of critical-size defects [6,8]. Long-term follow-up studies indicate favorable safety and durable implant outcomes when SVF is combined with osteoconductive scaffolds, further confirming that pro-angiogenic support and trophic factors enhance osteogenesis. Recent preclinical work also highlights the role of improved vascularization and matrix maturation with engineered SVF formulations [9].

In cartilage and knee OA, a growing clinical literature reports improvements in pain and function after intra-articular injections of fresh SVF or SVF-enriched products; imaging and small trials suggest structural benefits in some cohorts, although randomized controlled evidence remains limited, and heterogeneity in SVF preparation complicates comparisons. Recent systematic reviews/meta-analyses support the positive effect of SVF-mediated anti-inflammatory signaling, which reduces the impact of catabolic mediators (MMPs/IL-1β) and plays a role in trophic support for chondrocytes, alongside providing symptomatic relief and some structural MRI signals indicating cartilage regeneration. Overall, it can be stated that SVF is potentially promising for symptomatic OA, yet standardized randomized trials with long-term structural endpoints are needed [10,11].

Anti-scar and plastic surgery uses (SVF-enriched fat grafting or tSVF injected into wounds/incisions) consistently show early improvements in scar thickness, vascularity, and subjective aesthetic metrics; meta-analytic data and randomized trials support reduced early hypertrophy and improved wound quality, with few safety signals reported to date. These anti-fibrotic effects are mediated by SVF-driven shifts in macrophage phenotype, a reduction in pro-inflammatory cytokine expression, the modulation of collagen deposits, and improved vascularization, which ultimately supports healthy remodeling [7,12,13].

Neural applications—peripheral nerve and spinal cord injury repair—represent an active and rapidly evolving area, yet such applications are mostly still at the preclinical stage [14]. Preclinical contusion and transection models treated acutely with SVF show improved sensorimotor recovery, reduced lesion cavitation, enhanced angiogenesis, and lower inflammatory markers [15,16,17]. Clinical works also emphasize the potential of bedside-compatible SVF protocols to be deployed early after injury while noting the need for dose-finding, standardization, and safety data in controlled human studies [18]. Notably, recent preclinical reports demonstrated meaningful functional gains when SVF was delivered soon after injury. Indeed, paracrine neurotrophic and neoangiogenic support, reduced inflammation by means of reduction of inflammatory cytokines, improved axonal regeneration, and muscle reinnervation (when combined with conduits/scaffolds) are the functional recovery mechanisms supporting promising preclinical translational data [19,20,21].

A mechanistic insight shows a common cascade of events. Under the influence of SVF cells, in all the different clinical indications, the early modulation of inflammation is the first critical step to control and inhibit the acute inflammatory response, which allows for better regeneration compared to fibrotic healing. The paracrine and trophic effects are then central in tissue restoration, supported by cell differentiation and secretome-induced extracellular matrix modulation, which further contribute to anti-scar effects, hence further shaping the environment for healing. Finally, angiogenesis and microvascular neoformation are recurring enabling features that ensure long-term tissue vital remodeling.

Notably, the use of smart scaffolds, biomaterials, and combination therapies is also gaining traction, as these methods enhance the regenerative outcome of SVF alone. Indeed, SVF is being increasingly paired with scaffolds to further increase cell retention, structural support, and delivery of trophic, angiogenic, and immunomodulatory cues [22,23]. Recent advances emphasize single-point-of-care approaches, i.e., one-stage approaches wherein freshly isolated SVF is seeded into or onto a scaffold during the surgical procedure. This reduces culture expansion, regulatory burden, and maintains the heterogeneity and potency of the SVF cell population. Natural scaffolds—such as collagen, hyaluronic acid, gelatin/GelMA, decellularized extracellular matrix, and fibrin—are especially favored as they mimic the native tissue microenvironment and support angiogenesis. Composite scaffolds (e.g., ceramic/hydroxyapatite for bone, polymeric supports for cartilage, and aligned conduits for nerve) provide additional mechanical integrity. Mechanistically, scaffolds do more than merely retain SVF at the lesion site. They protect the cells; enable nutrient diffusion initially; modulate the immune cell infiltration; support pro-angiogenic growth factor release; and, in some configurations, also direct cell phenotype (e.g., favoring anti-inflammatory macrophage phenotypes, promoting pericyte/endothelial differentiation). Modular designs of scaffold porosity, stiffness, degradation kinetics, and surface cues are now being adjusted to match tissue type (e.g., bone vs. cartilage vs. dermal vs. neural) [3,22,23,24].

Though SVF is being widely used with promising clinical results, a critical analysis still demonstrates gaps that need to be addressed. In vitro and in vivo studies most commonly use enzymatic digestion methods to extract SVF from donor tissues, while clinical practice is almost only based on bedside extraction via mechanical systems for immediate autologous applications. Rare exceptions are those cases where SVF is extracted and expanded in certified cell factories, an approach that makes a simple, fast, and cheap procedure very expensive, complex, and slow. Heterogeneity in preparation methods (enzymatic vs. the wide variety of mechanical systems on the market, cell yield and composition, etc.) still makes comparisons difficult [11]. A side effect of this issue is also connected to the fine mechanistic understanding and the transability of preclinical evidence into clinical models [4]: which subpopulations in SVF are essential for what effect (e.g., anti-inflammatory macrophages vs. pericytes vs. endothelial progenitors) is not always crystal clear. Similarly, key aspects of dosing, timing, and cell yield variability are widely reported in the literature, and they represent a core pillar for safety first and foremost, as well as robustness in translation into clinics and for regulatory purposes. This further leads to numerous cases of less robust, high-scale standardization and regulatory compliance: indeed, high statistical significance in translation to large clinical trials, standardized dosing, safety, and cell tracking remains relatively limited, necessitating more long-term clinical assessments of structural regeneration and result durability over multiple years. Studies comparing SVF with and without specific scaffolds are still lacking as well, yet they represent an essential step towards safe standardization and assurance of effective and long-lasting safe therapies.

## 2. Clinical Perspectives and Future Directions

Recent clinical and translational studies increasingly position stromal vascular fraction (SVF) as a pragmatic, next-generation cell-based therapy with realistic potential for broader clinical adoption. Over the last five years, the literature has shifted from proof-of-concept applications toward attempting to prove SVF as a point-of-care, minimally manipulated, autologous biologic capable of addressing unmet clinical needs where conventional treatments remain limited or invasive. Emerging data suggest that SVF may be particularly well suited for early-intervention strategies, combination therapies with biomaterials, and indications requiring rapid immunomodulation and angiogenic support rather than long-term cell engraftment. From a regulatory and translational standpoint, mechanically isolated SVF aligns favorably with current frameworks for minimal manipulation, facilitating bedside use while avoiding ex vivo expansion. Ongoing efforts in standardized processing, potency assays, and indication-specific dosing are expected to enable more robust randomized trials, which will be critical to validate durability, safety, and cost-effectiveness. Collectively, these advances suggest that SVF is transitioning from an experimental regenerative adjunct toward a clinically actionable platform technology, especially in musculoskeletal, reconstructive, and neurotrauma applications.

However, to advance the field, we urgently need large, well-designed randomized controlled trials with standardized cell processing protocols, long-term follow-ups, and deeper mechanistic studies dissecting which cellular components mediate which effects. Such rigorous data would allow SVF to finally shift from a “promising experimental therapy”, with good results, to a standard-of-care therapy in selected indications, with reliable and robust results, which is especially important when considering that many current treatments are either inadequate or invasive, or even non-existent, as is the case with spinal cord injuries [25].

## 3. Discussion

Taken together, recent advances point towards the use of stromal vascular fraction as a biologically robust yet clinically pragmatic regenerative strategy whose strength lies in its cellular heterogeneity, paracrine activity, and compatibility with point-of-care workflows. The growing body of evidence from the last five years underscores that SVF efficacy is not confined to a single tissue or indication, but rather reflects a conserved, mechanism-driven response centered on immunomodulation, angiogenesis, and matrix remodeling. At the same time, contemporary studies highlight that successful clinical translation will depend less on increasing biological complexity and more on harmonizing processing methods, defining clinically meaningful endpoints, and integrating SVF within standardized surgical and biomaterial-assisted protocols, bearing in mind the key aspects of safety and efficacy, also within the regulatory framework of reference. Within this framework, it becomes possible to distill a set of core concepts that are progressively recorded across indications and studies, providing a concise rationale for the key take-home messages that follow:

1. SVF represents a pragmatic autologous approach to regenerative medicine across different clinical indications;
2. SVF has a paracrine and immunomodulatory mode of action;
3. The advantage of point-of-care is given by one-step SVF administration to preserve heterogeneity and avoid ex vivo expansion;
4. Synergy with scaffolds and biomaterials is enhancing SVF effects by improving cell retention, microvascolarization, and mechanical integration;
5. There is an urgent need for standardized isolation methods, potency assays, and randomized trials to confirm durability and clarify optimal dosing and timing.

## 4. Conclusions

Overall, the simple, fast, and cheap bedside use of SVF might constitute a unifying and promising therapeutic strategy that is undoubtedly appealing across multiple regenerative medicine disciplines, with an increasingly positive trend in clinical results. SVF has the capacity to provide trophic support and, hence, to establish a pro-regenerative environment at the graft or injury site, which results from the combination of direct differentiation of the stem population and the paracrine effects (cytokines, interleukins, growth factors, extracellular vesicles, etc.). The pathway to make SVF a consolidated clinical practice is still long, yet here, we placed emphasis on the *fil rouge*, weaving its properties together to address seemingly different clinical applications. SVF exerts effects through different mechanisms, remodels the extracellular matrix, and supports endogenous repair, often enhancing the effects of scaffolds, biomaterials, and surgical techniques [3,25].

## Data Availability

No new data were created or analyzed in this study.
